# Changes in BMI and Fat Mass and Nutritional Behaviors in Children Between 10 and 14 Years of Age

**DOI:** 10.3390/nu17071264

**Published:** 2025-04-03

**Authors:** Katarzyna Ługowska, Elżbieta Krzęcio-Nieczyporuk, Joanna Trafiałek, Wojciech Kolanowski

**Affiliations:** 1 Faculty of Medical and Health Sciences, University of Siedlce, 08-110 Siedlce, Poland; katarzyna.lugowska.zdoz@uws.edu.pl (K.Ł.); elzbieta.krzecio-nieczyporuk@uws.edu.pl (E.K.-N.); 2 Institute of Human Nutrition Sciences, Warsaw University of Life Sciences, 02-787 Warsaw, Poland; joanna_trafialek@sggw.edu.pl; 3 Faculty of Health Sciences, Medical University of Lublin, 20-400 Lublin, Poland

**Keywords:** body mass index, children, fat mass, nutritional behaviors, obesity

## Abstract

**Background/Objectives**: Unhealthy nutritional behaviors and excess body weight constitute a serious challenge for public health in children and adolescents. The aim of this study was to examine changes in body mass index (BMI), body fat mass (FM), and nutritional behaviors in the same group of children during a 4-year observation between 10 and 14 years of age including the period of the COVID-19 pandemic. **Methods**: BMI and FM using bioelectrical impedance were assessed. To assess nutritional behavior, a questionnaire on eating behavior was used. The study was carried out in a group of 250 children, starting from the age of 10 and finishing at the age of 14. The measurements were collected in the years 2017 and 2021. The results were compared and analyzed. **Results**: Excessive BMI (overweight and obesity) was more often found in girls (28.29%) than boys (23.63%), while normal body weight was more often found in boys (65.76%) than girls (60.96%). Between the initial and final assessments, the percentage of children with normal body mass decreased from 65.65% to 61.07%. Excessive BMI (overweight and obesity) increased from 27.09% to 29.50% in girls, and from 21.26% to 26.00% in boys. The mean percentage of FM was higher in girls than boys (23.17% vs. 16.20%, respectively). The mean FM decreased from 17.80% to 14.60% in boys and increased from 21.77% to 24.57% in girls. Poor nutritional behaviors were observed in 20.35% of children, more often in boys (22.25%) than in girls (18.50%). Between the initial and final assessments, an increase in the mean consumption of fruit, whole-grain bread, and milk was noted. These were products that should be consumed more often to demonstrate a healthy diet. However, the consumption of products that should be limited for a healthy diet, such as fried flour dishes, fried meat dishes, fatty cheeses, butter, fast food, sweets, and carbonated drinks, also increased. Boys more often than girls consumed red meat and poultry meat, eggs, butter, and fast food, while girls more often than boys consumed fruit, vegetables, yogurts, cottage cheese, wholemeal bread, fruit, and sweets. **Conclusions**: Children usually showed moderate nutritional behavior. After four years, there was a significant increase in the consumption of fruit and whole-grain bread, i.e., products that should be consumed as part of a healthy diet, as well as fried flour and meat dishes, fatty cheeses, butter, fast food, and sweets, i.e., products whose consumption should be limited. With age, the percentage of children showing unfavorable nutritional behaviors and excessive body weight increased. More extreme levels of overweight and obesity and higher body fat contents were found in girls than boys. Although girls’ nutritional behaviors were healthier, they were at a higher risk of excessive body weight. Increased promotion of a healthy diet and regular monitoring of body fat content in school-aged children is strongly recommended.

## 1. Introduction

Children and adolescents are especially at risk of obesity and unhealthy nutritional behaviors [1]. A 2024 World Health Organization (WHO) report indicated that nearly one-quarter of adolescents are overweight or obese [2,3]. The COVID-19 pandemic and related lockdowns have contributed to an increase in this unfavorable trend. Estimates of the global level of overweight and obesity suggest that the incidence of being overweight occurs fastest in children and adolescents [1,4]. For example, Poland is one of the countries in which adolescents gain weight at a very rapid rate [5]. In Poland, one-third of school-aged children are overweight or obese [6]. A healthy diet and regular physical activity (PA) are essential in preventing and controlling excess body weight and related noncommunicable diseases [4].

Excessive body weight and body fat increase the risk of hyperlipidemia, type 2 diabetes, hypertension, decreased circulatory and respiratory capacity, reduced muscle strength and physical fitness, and rapid fatigability [1,2]. The main objective of an obesity treatment and prevention program for school-aged children should not only be to reduce body weight but also to normalize what is a healthy amount of adipose tissue. All obesity-prevention strategies start with a healthy diet and increased PA [7,8,9,10,11,12]. Body mass index (BMI), which determines the proportion of body weight to height, is used to assess nutritional status, but it does not provide information on the content of body fat mass (FM) [13,14,15]. In the assessment of adipose tissue content, dual-energy X-ray absorption is considered the gold standard [16]. This method is stationary and is not suitable for field studies outside of the laboratory. Therefore, the most commonly used method for assessing FM in school studies in children and adolescents is skin folds and bioelectrical impedance analysis (BIA) [17,18]. The content of fat tissue is traditionally estimated by frequently used cheap skinfold measurements and a calculation using a special mathematical algorithm. However, BIA gives more precise values. BIA does not directly measure FM but uses algorithms that take resistance and reactance parameters into account to estimate it. Children and adolescents change their body composition with age. During puberty, a significant increase in FM in girls and muscle mass in boys is often observed [19,20,21].

Nutritional recommendations for children and adolescents are presented in graphic form as a healthy eating plate that shows the correct composition of a diet [19,22]. According to the recommendations, the diet of school-aged children should be based on vegetables and fruits, low-fat dairy products, eggs, whole-grain products, thick groats, and other foods with high nutrient density. Red meat should be replaced with lean poultry and wheat bread with whole-grain and coarse groats rich in dietary fiber. It is important to limit fried meat or flour dishes as well as fatty foods [19]. The basic drink should be water.

Many studies demonstrated that adolescents’ dietary habits usually change unfavorably compared to childhood [23,24,25]. Unfortunately, an increased consumption of highly processed foods rich in fat, sugars, and salt, an excessive consumption of sweets, and an excessive consumption of carbonated drinks are often observed [26]. Furthermore, differences in dietary behaviors between girls and boys are also visible [26,27]. Girls are more aware and make better nutritional choices during puberty than boys [26,27]. They show a greater preference for whole-grain products, fruits, vegetables, dairy products, lean meat, and thick groats [25,26,27]. Boys drink carbonated and sugar-sweetened beverages and eat sweet snacks more often than girls [27]. Girls eat more regular meals and drink more water than boys [25,26,27]. Despite the observed differences in gender, a general increase in unfavorable nutritional behaviors and obesity has been noted.

Unfortunately, studies have shown many irregularities in the nutrition of children and adolescents, including a low consumption of vegetables, fruit, fish, and whole-grain products, with a high consumption of sweets, salty snacks, and sweet drinks [28,29]. The recommended healthy diet for children and adolescents is most like the Mediterranean diet. Numerous epidemiological studies have emphasized the properties of the Mediterranean diet that help prevent noncommunicable diseases and maintain a healthy weight [30,31,32].

There are studies that analyze the body composition and nutritional behaviors of school-aged children, but there are no complete or long-term analyses comparing school-aged girls and boys, considering an age range. The increase in unfavorable nutritional behaviors and the obesity epidemic led us to study body mass and body fat content in relation to the nutritional behaviors of girls and boys. It is also believed that the lockdowns during the COVID-19 pandemic have had a negative effect on the above parameters. Our working hypotheses assume that there are differences in nutritional behaviors, BMI, and FM within the sexes. With age, an awareness of nutritional behaviors increases. Hormonal changes in adolescence affect the increase in body weight, particularly in girls. The increase in obesity allows us to assume that the problem of excess body weight will be greater in older children. The COVID-19 pandemic has undoubtedly influenced the increase in unfavorable nutritional behaviors, BMI, and FM among school-aged children. The aim of this study was to examine changes in BMI, FM, and nutritional behaviors in the same group of children during a 4-year observation between 10 and 14 years of age including the period of the COVID-19 pandemic.

## 2. Materials and Methods

### 2.1. Participants

This was an observational cohort study that evaluated body fat, BMI, and nutritional behaviors in children. The study was performed on the same group of children born in 2007 who attended 6 primary schools in Siedlce, a medium-sized city in central Poland. The initial phase of the study was conducted in 2017 (the children were approximately 10 years old) and the final stage was conducted in 2021 (the children were approximately 14 years old). Each class had 22–25 children. The children who participated in the study came from similar backgrounds and had similar socioeconomic statuses. A total of 304 participants, including 142 girls and 162 boys, agreed to take part in the study. The sampling strategy was based on convenience sampling, and therefore, the selected groups were not equal. The sample size was justified by power analysis in G*power software (version 3.1.9.7; Universität Kiel, Kiel, Germany) with a type i error rate of 0.05 and 80% statistical power [33]. Overall, the analysis indicated that a total of 250 participants is sufficient to observe significant effects (Cohen’s d = 0.80). The study consisted of two parts. The first part was a survey of nutritional behavior and the second part was anthropometric measurements to determine BMI and body composition analysis to estimate FM.

The children were informed about the confidentiality of the results and the purpose of the study before they began the study. The project received a positive response from the management of each school, the teachers, and the parents. All parents or guardians gave written consent for their children to participate in the study. The parents declared that they would prepare the child for the measurements in accordance with detailed guidelines. The positive impact of parental involvement in preparing children for the measurements has been previously demonstrated [34]. The study was approved by the Research Ethics Committee at the University of Siedlce (No. 2/2016). The inclusion criteria for the study were as follows: (1) good health (no newly diagnosed chronic diseases, injuries, or wounds and good well-being); (2) age 10 years at the beginning of the study and 14 years at the end of the study; (3) consent from the child and their parents to participate in the study; (4) correctly completed questionnaire; (5) correctly performed anthropometric measurements and body composition analysis; (6) the child did not follow a special diet that could potentially affect the final result; (7) no injuries or wounds during the measurement session; and (8) no pacemaker implanted, which could affect the BIA measurement result.

The team assumed that children who joined a given class from another school during the study would be subjected to anthropometric measurements and an analysis of nutritional behaviors after meeting the above criteria. However, children who dropped out of a given class or school during the study were not subject to further analysis due to the lack of the possibility to perform measurements.

The study was conducted in different schools following the same procedure. The research team performed anthropometric and body composition measurements and supervised the completion of the questionnaire on nutritional behaviors. Before the proper studies, in 2017, pilot studies were conducted on 10–11-year-old children to check the correctness of the adopted assumptions and methodology. The pilot study was conducted on a group of 43 children (24 girls; 19 boys) and was conducted from May to June. The aim of this pilot study was to examine the procedure used to ensure the children completed the questionnaire and take anthropometric and body composition measurements. The pilot study was planned and conducted properly. The methods used in the pilot study were accepted in the main study.

### 2.2. Procedure

The measurements were carried out by a qualified and trained team of dietitians. The measurements were taken on two dates: the initial session in September 2017 and the final session in September 2021. The measurements were performed using the same equipment used in all schools. BMI and FM values were interpreted based on percentile charts that were appropriate for age and gender [26,30,35]. Anthropometric measurements and body composition analysis were carried out according to the methodology described in our previous works [9,10,21,36].

Nutritional behaviors were assessed using an anonymous survey based on the Questionnaire of Eating Behavior (QEB) from the Polish Academy of Sciences [37]. The results were compared to nutritional recommendations [13,22,31,32]. Children completed the same questionnaire in 2017 and 2021. The results showing the nutritional behaviors of children at the age of 14 were published in our previous article [36].

The anthropometric and body composition measurements were performed between 10:00 and 11:00 a.m. in the gym in the presence of a teacher, following the guidelines necessary for the accurate measurement of body weight, height, and body composition analysis [38]. Parents were instructed to not allow their children to eat heavy and large meals after 9:00 p.m. the evening before the measurement, and on the day of the measurement, children were advised to eat a light breakfast. The research team recommended that the last organized physical activity, aside from necessary household chores, should be performed at least 12 h before the measurement. Before the measurement, children were asked not to eat or drink until the measurement was complete [16,17].

### 2.3. BMI Calculation

Body weight (kg) and height (cm) were measured, which allowed for the BMI calculation. Body weight and height were measured twice, and the results were averaged. BMI was calculated as body weight divided by height (kg/m^2^) [39,40]. BMI percentile charts were used to interpret the results [14,35]. Measurements were carried out according to a standard procedure. Height was measured with a Seca 214 stadiometer (Seca GmbH & Co. KG, Hamburg, Germany) in an upright position with an accuracy of 1 cm. Body weight was measured using a Tanita SC-240MA device (Tanita Cooperation, Tokyo, Japan) with an accuracy of 0.1 kg [39,41,42]. According to the criteria, overweight was considered as BMI ≥ 85th percentile, obesity as BMI ≥ 95th percentile, and underweight as BMI ≤ 10th percentile [14,35]. Previous works described procedures for measuring body weight and height [9,10].

### 2.4. Fat Mass Estimation 

BIA measurements were performed using a Tanita SC-240MA body composition analyzer. The analysis allowed for the estimation of FM. The collected results were only indicative since the analyzer did not provide parameters of resistance, reactance, impedance, and phase angles [39,41,42]. The measurements were performed according to the standard protocol and manufacturer’s recommendations, without shoes or socks, with clean and dry feet, and wearing light clothing. Participants remove metal jewelry or clothing containing metal zippers, snaps, or buttons from their bodies. The children were asked to avoid exertion and excessive fluid intake before the test, to stand for at least 5 min before the measurement to distribute tissue fluids, to empty their bowels and bladder at least 30 min before the measurement, and to perform the measurement in a standing position. Before each measurement, the surfaces of the analyzers were cleaned. After each surface had dried, the participants were instructed to stand straight on the four electrodes with bare feet, their arms moved away from the body, and their legs slightly apart [39,41,42].

The detailed procedure for preparing children for body composition analysis was described in previous works [21]. Percentile charts were used to interpret the results [18]. The recommendations of McCarthy et al. (2006) [18] were referred to for the FM values. The 2nd and 85th percentiles indicated children with deficient and an excess of adipose tissue, respectively, and the 95th percentile indicated obese children. Body fat percentage indicated the ratio of fat to total body mass, while the fat mass in kg represented the actual body fat mass.

### 2.5. Questionnaire

The declared nutritional behaviors were examined using an anonymous questionnaire. There were 31 close-ended questions with one possible answer to choose from in the survey. Children completed the same survey in the initial (2017) and final (2021) sessions. The questionnaire was validated in a pilot study. Children were not time-limited. Participants received a short set of instructions before they started the survey.

The questionnaire asked about the frequency of consumption of food products, dishes, and beverages. The first group of questions focused on foods that should be consumed frequently in a healthy diet (products with beneficial health properties), including milk, yogurts, cottage cheese, thick groats, oatmeal, whole-grain pasta, whole-grain bread, fish, poultry dishes, fruits, vegetables, legume dishes, and eggs. The second group of questions focused on foods that should be consumed moderately or low in a healthy diet, including white rice, white bread, fine-grain groats, flour dishes, fried meat dishes, lard, butter, fatty cheeses, red meat dishes, cold cuts, sausages, frankfurters, canned meats, sweetened drinks, energy drinks, fast food, and sweets. Moreover, the questionnaire included questions about the regularity of breakfast consumption, snacking between meals, and the most frequently chosen liquids to drink. The results were compared with the healthy diet recommendations for children and adolescents [19,22,31,32].

Nutritional behaviors were evaluated on a three-point scale of very good, moderate, and poor. A very good score was given when whole-grain products, thick groats, vegetables, fruits, milk and fermented dairy products (e.g., yogurts), cottage cheese, and eggs were eaten every day. The score was also given when poultry meat, red meat, fish, legumes, butter, fatty cheeses, wheat bread, wheat pasta, small groats, fried flour dishes, and fried meat dishes were eaten several times a week. A very good score was also given if sweets, fruit juices, carbonated and energy drinks, cold cuts, sausages, frankfurters, lard, canned meat, and fast food appeared only several times a month or not at all.

A moderate score corresponding to moderate nutritional behaviors included the daily consumption of red meat, legumes, butter, fatty cheeses, wheat bread, wheat pasta, small groats, and fried flour or meat dishes, as well as the consumption several times a week of whole-grain products, coarse groats, whole-grain pasta, oatmeal, vegetables, fruit, milk and fermented products, low-fat cottage cheese, fish, and eggs. A moderate score was also given if sweets, fruit juices, carbonated and energy drinks, fatty cold cuts, sausages, frankfurters, lard, canned meat, and fast food appeared several times a week.

A poor score corresponding to unhealthy nutritional behaviors included the daily consumption of sweets, fruit juices, carbonated and energy drinks, fatty cold cuts, sausages, frankfurters, lard, canned meats, and fast food. A poor score was also given if the diet included only one or several times a week the desired whole-grain products, such as thick groats, whole-grain pasta, oatmeal, vegetables, fruits, milk, fermented milk products, cottage cheese, poultry or fish, and eggs. Furthermore, it was given when red meat, cold cuts, butter, fatty cheeses, wheat bread, wheat pasta, small groats, fried flour dishes, and fried meat dishes were consumed daily. The detailed procedure related to the conduct of the research and analysis was presented in our earlier work [36]. The results of the research conducted on 14-year-old children were presented in our earlier work [36].

### 2.6. Statistical Analysis

The statistical calculations were performed using Microsoft Excel 365 (Microsoft, Corp., Washington, DC, USA) and Statistica 13 (Stat Soft, Krakow, Poland). The level of statistical significance was set at α ≤ 0.05. The mean level according to gender and age was calculated.

The *t*-test, Shapiro–Wilk test, Mann–Whitney U test, one-way ANOVA, χ^2^ test, and analysis of variance were used for statistical analysis of the results. The normality of the distribution was assessed using the Shapiro–Wilk test and the assumption of homogeneity of variance was verified with Levene’s test for *p* > 0.05. The Mann–Whitney U test analyzed parameters without a normal distribution.

The mean, median, standard deviations, and 95% confidence intervals were calculated to assess body fat content according to the different sex and age profiles. The effect size (ES) for the mean for FM of children was calculated based on Cohen’s d. The threshold values for ES statistics were as follows: >0.2 low, >0.5 medium, >0.8 high, and >1.3 very high [43].

The χ^2^ test was used in the analysis of nutritional behavior. The strength of the association between sex and nutritional behaviors was assessed using Cramer’s V (VC), which is based on Pearson’s χ^2^ statistic and has an inclusive value from 0 to 1, ranging from no association at 0 and increasing to the full association at 1, respectively.

Differences between the baseline and final mean values of FM and BMI category variables and anthropometric measurements, and BMI and FM categories, were determined using the one-way analysis of variance. The Student *t* test was used to compare mean values between baseline and final BMI measurements.

Additionally, a principal component analysis (PCA) was undertaken. The PCA used the average results of the initial and final measurement sessions for the BMI, FM, favorable nutritional behavior, and unfavorable nutritional behaviors, for both girls and boys.

Multivariate cluster analysis was used to assess the influence of gender on adverse nutritional behaviors, BMI obesity, and FM obesity. Mean values were used for the above calculations. The analysis allowed for the drawing of a tree diagram in which the values obtained by girls and boys were most similar in a specific cluster. Cluster validation was conducted using intrinsic measures such as matrix and Euclidean distance. Ward’s method, such as a hierarchical clustering method, was used to create groups in which variance within the groups is minimized.

## 3. Results

### 3.1. Group Characteristics

During the initial phase of the study, 304 children (with a mean age of 10.27 years) were included (142 girls; 162 boys). In the survey, 278 questionnaires were included (127 girls; 151 boys). Nearly 6% of the questionnaires from boys and 4% of the questionnaires from girls were excluded because they were not filled out correctly. The first stage of anthropometric measurements was conducted among 245 children (118 girls; 127 boys). About 17% of girls and 22% of boys did not participate in the measurements, despite initial consent.

In the final stage of the study, consent was obtained from 236 parents and adolescents. However, in the final analysis, the results obtained from 213 participants (106 girls; 107 boys) were included. The reason for the exclusion was the participant’s absence on the day of measurement. This was 9.75% of children.

### 3.2. Anthropometric Indicators

Table 1 presents the mean height, body mass, and BMI at the beginning and end of the study. Compared to the initial period, the average height of the children increased on average by 24.24 cm (girls increased by 22 cm; boys increased by 26.48 cm; *p* = 0.001). Boys were taller than girls (girls were 153.67 cm; boys were 156.52 cm; *p* = 0.087). The weight gain in the entire group was on average 22.08 kg (girls gained 19.73 kg; boys gained 24.43 kg; *p* = 0.001). On average, girls had a lower body mass than boys (47.32 kg vs. 50.36 kg, respectively; *p* = 0.062). The mean increase in the BMI of girls was 2.78 kg/m^2^ from 18.28 to 21.06 and of boys was 3.15 kg/m^2^ from 18.38 to 21.53 (*p* = 0.001). On average, girls had a slightly lower BMI compared to boys (19.67 vs. 19.95 kg/m^2^, respectively; *p* = 0.071). The girls and boys had similar BMI percentiles at the beginning (50th) and at the end of the study (75th). There was an increase in the mean BMI percentile from 50th to 75th in both girls and boys (*p* = 0.731).

### 3.3. BMI

A more detailed data set can be obtained by determining the specific BMI categories in the group. On average, about 15% of children were overweight (15.76% of girls; 13.12% of boys; *p* = 0.001) and 11.51% were obese (12.53% of girls; 10.50% of boys; *p* = 0.000). A normal body weight was noted in 63.36% of children (Table 2). An excessive body weight (overweight and obesity) was significantly more common in girls (28.29% of girls; 23.63% of boys; *p* = 0.024). A normal body weight occurred more often in boys (60.96% of girls; 65.76% of boys; *p* = 0.031). The mean percentage of underweight children was similar in both sexes (10.61% of boys; 10.74% of girls; *p* = 0.671).

**Figure 1:**
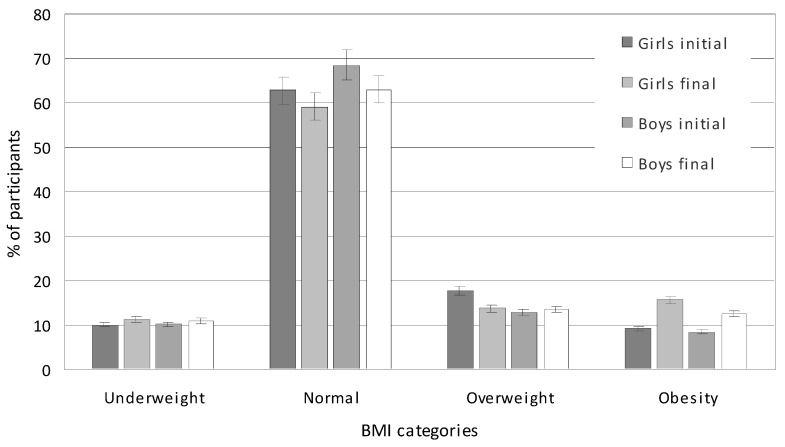
BMI categories of studied children in the initial (2017) and final (2021) measurement sessions.

After four years, the average percentage of children with normal body weight decreased from 65.65% to 61.07% (*p* = 0.001). In girls, this percentage decreased from 62.75% to 59.15% (*p* = 0.023); in boys, it decreased from 68.52% to 63% (*p* = 0.000) (Figure 1). Throughout the study period, girls were more likely to be overweight or obese than boys. Between the initial and final measurement, a significant increase in the percentage of children with excessive body weight (overweight and obesity) was noted in girls from 27.09% to 29.50% (an increase of 2.41%; *p* = 0.070) and in boys from 21.26% to 26.00% (an increase of 4.74%; *p* = 0.035). The mean percentage of underweight children increased slightly from 10.71% to 11.17% (*p* = 0.091).

### 3.4. Fat Mass

On average, girls had a higher FM than boys over the entire study period (23.17% of girls; 16.20% of boys; *p* = 0.011). Compared to the initial measurement, in boys, the mean FM decreased from 17.80% to 14.60% (*p* = 0.040), and in girls, it increased from 21.77% to 24.57% (*p* = 0.020) (Table 3). Girls had a higher FM expressed in kilograms than boys (11.80 kg and 8.68 kg, respectively; *p* = 0.001). Compared to the initial measurement, in girls, the FM increased by an average of 6.15 kg, and in boys, it increased by an average of 2.38 kg (*p* = 0.004).

A total of 67% of children (21.85% of girls; 19.49% of boys; *p* = 0.001). Obesity FM was found in 12.45% of children (12.60% of girls; 11.89% of boys; *p* = 0.074). Normal FM was found in 59.46% of children (57.56% of girls; 61.36% of boys; *p* = 0.081). Underweight FM occurred in 8.24% of children (7.98% of girls; 8.51% of boys; *p* = 0.091) (Table 4).

**Figure 2:**
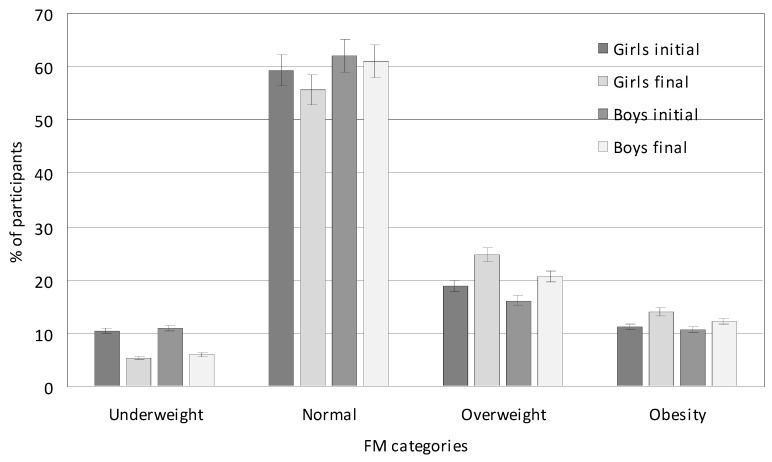
FM categories of studied children in the initial (2017) and final (2021) measurement sessions.

Compared to the initial period, the percentage of children with excessive FM (overweight and obesity) increased from 28.52% to 35.88% (*p* = 0.001). Similarly to BMI, girls had a higher average percentage of excessive FM than boys throughout the study period. Between the initial and final measurements, there was a significant increase in the percentage of children with excessive FM (overweight and obesity): in girls, it increased from 30.15% to 38.75% (an increase of 8.60%; *p* = 0.047), and in boys, it increased from 26.88% to 33.02% (an increase of 6.14%; *p* = 0.035). The percentage of children who had overweight FM increased from 17.59% to 22.76%. This was especially evident in girls where the percentage of overweight FM increased from 18.95% to 24.75% (*p* = 0.057) and to a lesser extent in boys—from 16.22% to 20.77% (*p* = 0.065) (Figure 2). The percentage of children with obesity FM increased from 10.93% to 13.12% (*p* = 0.095). In girls, this percentage increased from 11.20% to 14.00% (*p* = 0.065), and in boys, this increased from 10.66% to 12.25% (*p* = 0.057). The percentage of children with normal FM decreased from 60.76% to 58.37% (*p* = 0.085). In girls and boys, the percentage decreased similarly from 59.33% to 55.80% and from 62.15% to 60.95% (*p* = 0.085). The percentage of children with underweight FM decreased from 10.75% to 5.73% (*p* = 0.025). In girls and boys, the percentage decreased similarly from 10.52% to 5.45% and from 10.99% to 6.03%, respectively (*p* = 0.001).

### 3.5. Nutritional Behaviors

The average declared frequency of food consumption during the entire study period is presented in Table 5. Between the initial and final sessions, a decrease in very good behaviors was noted. On average, 23.25% of children had very good nutritional behaviors during the entire study period. A higher percentage of children showing very good nutritional behaviors was noted in girls than in boys (26.30% and 20.25%, respectively; *p* = 0.071). At the beginning of the study, 28.40% of girls and 20.25% of boys received a very good assessment (*p* = 0.041), and at the final stage, 24.30% and 20.20%, respectively (*p* = 0.069). Moderate nutritional behaviors were noted in 57.50% of children (56.40% of girls; 58.20% of girls; *p* = 0.097). The percentage of girls showing moderate nutritional behaviors was the same at the beginning and at the end of the study, i.e., 56.40%, while in boys, it decreased from 64.25% to 52.30% (*p* = 0.041). Poor nutritional behaviors were noted in 20.35% of children and were more common in boys (18.50% of girls; 22.25% of boys; *p* = 0.096). It was observed that with age, the percentage of children with unfavorable nutritional behaviors increased. At the beginning of the study, poor nutritional behavior was shown in 16.30% of girls and 16.25% of boys (*p* = 0.192), and at the final stage, 20.20% and 28.40%, respectively (*p* = 0.092).

On average, most girls showed very good nutritional behaviors concerning the consumption of milk (69.72%), yogurts (61%), vegetables (57.48%), and fruit (54.55%). Energy drinks were not consumed at all by 81.75% of girls, 64.25% did not consume canned meat, and 93.75% did not consume lard. Just 40.00% of girls drank carbonated drinks only one–three times a month and 21.25% never at all. Unfortunately, 38.75% drank them more frequently. Moderate nutritional behaviors included the consumption of poultry meat (40.50%), red meat (49.52%), fish (53.36%), and eggs (58.65%) once a week. Whole-grain bread and cold cuts and sausages were consumed several times a week (38.84% and 60.79%, respectively), and once a week, coarse-grained groats (52.71%), fine-grained groats (54.04%), and cottage cheese (29.25%) were consumed. The everyday consumption of wheat bread was declared by 69.31% of girls. Fast food was consumed one–three times a month by 52.43% of girls. Poor nutritional behaviors in girls included eating sweets every day (42.25%) and drinking fruit juices only once a week (46.00%). Poor nutritional behaviors also included the high consumption of fatty cheeses (54.38%) and fried meat dishes and fried flour dishes (66.75%). Also, 43.75% of girls only ate legume dishes one–three times a month. The everyday consumption of butter was declared by nearly 46.50% of girls.

Very good nutritional behaviors in boys concerned the daily consumption of fruit (46.26%) and vegetables (46.53%). Milk was consumed several times a week by 69.71% of boys and yogurt by 65.25%, including those who consumed it every day. Canned meat was not consumed by 64.25% of boys and lard was not consumed by 90.25%. The majority of boys (73.75%) did not drink energy drinks at all.

Moderate nutritional behaviors in boys included consuming poultry meat (34.99%), red meat (44.97%), fish (52.74%), eggs (58.97%), and whole-grain bread (33.62%). Among others, coarse-grained groats (55.74%) and fine-grained groats (54.04%) were consumed once a week. Several times a week, cold cuts and sausages (75.61%) and cottage cheese (44.25%) were consumed. The daily consumption of wheat bread was declared by 79.50% of boys. Fast food was consumed one–three times a month by 44.36% of boys.

Poor nutritional behaviors concerned the daily consumption of sweets (36.25%) and butter (48.25%). Fast food was consumed once a week by 48.05% of boys, and 46.25% of boys drank carbonated drinks once a week. Poor nutritional behaviors included the consumption of fruit juices only once a week (56.50%). Low consumption of legumes, only one–three times a month, was noted (46.50%), and 35.00% did not eat the above products at all.

Most often, the children ate four meals a day (37.34% of girls; 40.51% of boys; *p* = 0.071). About 30% of children ate five meals (32.04% of girls; 27.87% of boys; *p* = 0.061). Almost 66% of participants ate breakfast every day (66.84% of girls; 63.83% of boys; *p* = 0.310).

Snacks between main meals were consumed by 87.10% of participants; boys snacked slightly more often than girls (86.07% of girls; 87.52% of boys; *p* = 0.730). Most often, participants ate fruits every day (54.00% of girls; 46.26 of boys; *p* = 0.101), as well as salty snacks (26.67% of girls; 24.50% of boys; *p* = 0.080) and sweets (42.25% of girls; 36.25% of boys; *p* = 0.315). Only 1.25% of children consumed vegetables between meals (0.51% of girls; 2.10% of boys; *p* = 0.187). The most popular drink was water (61.26% of girls; 57.62% of boys; *p* = 0.094). Tea without sugar was drunk by 16.45% of girls and 10.96% of boys (*p* = 0.061).

Daily milk consumption was more often found in girls than boys (39.31% of girls; 27.95% of boys; *p* = 0.046), as well as yogurts (20.75% vs. 15.50%, respectively; *p* = 0.033) and cottage cheese (12% of girls; 10.75% of boys; *p* = 0.871), while the consumption of fatty cheeses was at a similar level for both (17.13% of girls; = 17.00% of boys; *p* = 0.130) (Table 5). The results showed that a high percentage of children, on average 74.40%, declared the consumption of wheat bread at least once a day, and this was more often boys than girls (79.50% vs. 69.31%, respectively; *p* = 0.034). The children rarely consumed whole-grain products, with only 15.39% of participants consuming them daily (18.68% of girls; 12.10% of boys; *p* = 0.010). Whole-grain bread was consumed several times a week by 22.16% of girls and 18.75% of boys (*p* = 0.092). Only 6.25% of participants consumed coarse-grained groats or oatmeal once a day (6.70% of girls; 5.83% of boys; *p* = 0.184). These products were consumed once a week by 52.71% of girls and 55.74% of boys (*p* = 0.071). More than half of the participants consumed white rice, fine-grained groats, and white pasta once a week (54.04% of girls; 54.22% of boys; *p* = 0.141). The frequency of the consumption of these products by girls and boys was similar (*p* = 0.291).

On average, boys reported more frequent consumption of poultry meat dishes than girls. They were consumed once a week by 40.25% of participants (40.50% of girls; 34.99% of boys; *p* = 0.021), and 25% consumed them every day (22.35% of girls; 26.80% of boys; *p* = 0.010). The children consumed poultry meat more often than red meat. Red meat was consumed most often once a week, on average by 47.24% of children (49.52% of girls, 44.97% of boys; *p* = 0.000). The consumption of red meat several times a week was declared by 16.01% of girls and 34.56% of boys (*p* = 0.000). Participants reported a high consumption of cold cuts and sausages. These products were consumed daily by 27.33% of girls and 41.37% of boys (*p* = 0.007). The children only occasionally consumed canned meat, and 65.00% of girls and 64.25% of boys did not consume canned meat at all (*p* = 0.694). A high consumption of fried dishes, both meat and flour, was noted, with 22.00% of children reporting to eat them every day (23.00% of girls; 21.00% of boys; *p* = 0.161) and 46% several times a week (43.75% of girls; 48.25% of boys; *p* = 0.810). Girls consumed the above products more often than boys (*p* = 0.681). About 58.80% of children ate eggs once a week, and 22.01% several times a week (19.04% of girls; 24.99% of boys; *p* = 0.004). The frequency of fish consumption was low. Once a week, 53.05% of children ate fish (53.36% of girls; 52.74% of boys; *p* = 0.841) and 26.33% ate fish once a month, while 17.08% did not eat fish at all.

The consumption of legumes was very low. Only 12.00% of girls and 12.50% of boys consumed them once a week, and about 45.12% one–three times a month (43.75% of girls; 46.50% of boys; *p* = 0.071). Legume dishes were not eaten at all by 35.10% of children (35.25% of girls; 35.00% of boys; *p* = 0.270). Girls consumed fruits and vegetables more often than boys. Vegetables were consumed daily by 57.48% of girls and 46.53% of boys (*p* = 0.021), and several times a week by 26.83% and 30.58%, respectively (*p* = 0.045). Fruit was consumed daily by 54.55% of girls and 46.26% of boys (*p* = 0.013).

Butter was the most frequently consumed fat. Every day, 46.50% of girls and 48.25% of boys consumed it (*p* = 0.090), and it was consumed several times a week by 27.75% and 23.00%, respectively (*p* = 0.061). The most frequently declared consumption of fast food was one–three times a month, declared by 52.43% of girls and 44.36% of boys (*p* = 0.031), while consuming once a week was declared by 35.62% and 48.05%, respectively (*p* = 0.002). Boys consumed fast food more often than girls (*p* = 0.016). The results showed a high consumption of sweets. Girls ate sweets more often than boys. Sweets were eaten daily by 42.25% of girls and 36.25% of boys (*p* = 0.040), and several times a week by 29.75% of girls and 25.50% of boys (*p* = 0.017). Lard was consumed very rarely. Only 6.10% of children consumed it consciously one–three times a month, and 92.00% declared that they did not eat it at all (girls 93.75%; boys 90.25%; *p* = 0.107).

Fruit juices were drunk more often than carbonated drinks by boys, and carbonated drinks more often than juices by girls. Participants usually consumed juices once a week (46.00% of girls; 56.50% of boys; *p* = 0.016). Juices were drunk one–three times once a month by 19.50% of girls and 16.25% of boys (*p* = 0.081). Sweetened carbonated drinks were drunk once a week by 28.00% of girls and 23.00% of boys (*p* = 0.101). Carbonated drinks were drunk one–three times a month by 38.50% of children (39.75% of girls; 37.25% of boys; *p* = 0.767). On average, boys were more likely to drink carbonated drinks than girls (*p* = 0.147). In turn, energy drinks were not drunk at all by 81.75% of girls and 73.75% of boys (*p* = 0.030), and 16.25% drank them only one–three times a month (15.50% of girls; 17.00% of boys; *p* = 0.083). Boys drank energy drinks more often than girls (*p* = 0.143).

**Figure 3:**
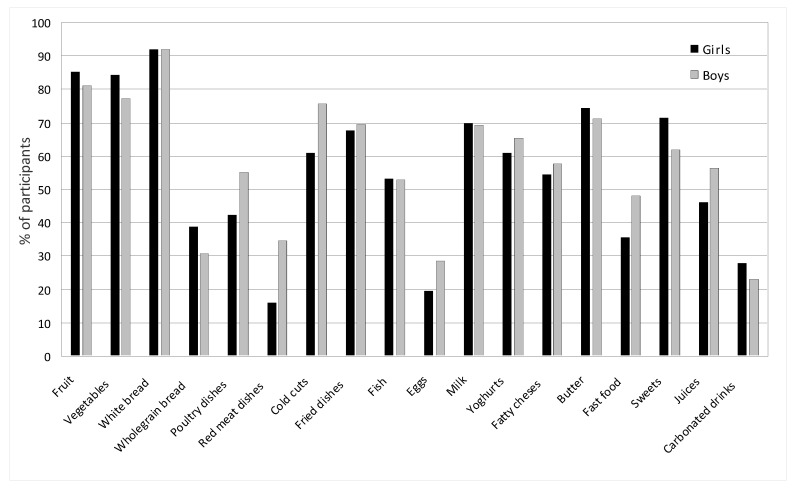
Average consumption of products and beverages among girls and boys. In the case of frequently consumed foods, such as milk, yogurt, fatty cheeses, wheat bread, poultry meat, cold cuts, fried dishes, fruit, vegetables, butter, and sweets, the values from the indications of several times a week and every day were summed. In the case of foods less frequently consumed, such as cottage cheeses, whole-grain bread, fish, white rice and pasta, eggs, fish, fast food, fruit juices, and carbonated drinks, the data from the indication of once a week were adopted.

Figure 3 shows the average consumption of products and drinks by children over the entire study period. In the case of frequently consumed foods, such as milk, yogurt, fatty cheeses, wheat bread, poultry meat, cold cuts, fried dishes, fruit, vegetables, butter, and sweets, the values from the indications of several times a week and every day were summed. In the case of foods less frequently consumed, such as cottage cheeses, whole-grain bread, fish, white rice and pasta, eggs, fish, fast food, fruit juices, and carbonated drinks, the data from the indication of once a week were adopted.

The products most frequently consumed by children were white bread (92% of girls; 91.96% of boys; *p* = 0.071); fruits (85.10% of girls; 81.16% of boys; *p* = 0.097); vegetables (84.31% of girls; 77.11% of boys; *p* = 0.004), milk (69.71% of girls; 69.35% of boys; *p* = 0.911), butter (74.25% of girls; 71.25% of boys; *p* = 0.101), cold cuts and sausages (60.78% of girls; 75.61% of boys; *p* = 0.031), fried dishes, both flour and meat (67.50% of girls; 69.50% of boys; *p* = 0.070), and sweets (71.50% of girls; 61.75% of boys; *p* = 0.022). The children were more likely to choose poultry than red meat (48.65% vs. 25.28%, respectively; *p* = 0.001), and white bread was also consumed more often than wholemeal bread (91.98% vs. 34.84%, respectively; *p* = 0.001). Milk was consumed more often than yogurt (69.53% vs. 63.12%, respectively; *p* = 0.041). Most often fish was consumed once a week by 53.36% of girls, and 52.74% of boys (*p* = 0.187). Similarly fast food (35.62% of girls; 48.04% of boys; *p* = 0.002). Juices were drunk more often than carbonated drinks (51.25% vs. 25.50%, respectively; *p* = 0.001). Boys more often chose juices (46% of girls; 56.50% of boys; *p* = 0.001), while girls chose carbonated drinks (girls 28% of girls; 23% of boys; *p* = 0.041).

### 3.6. Analysis of the Variability of Nutritional Behaviors

**Figure 4:**
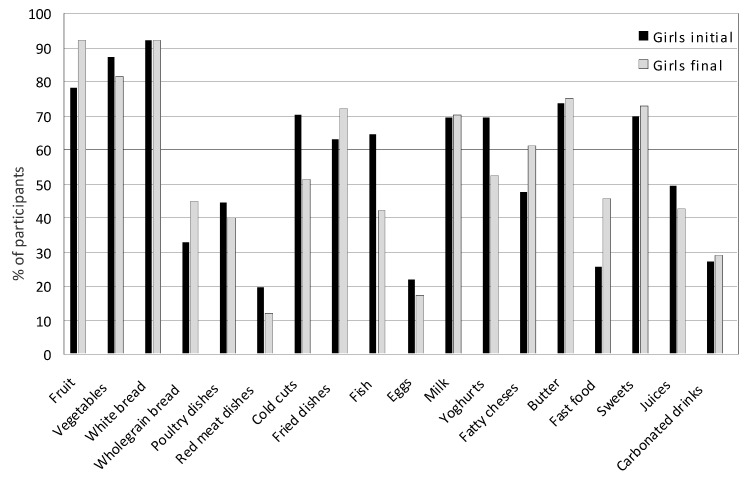
The average consumption of selected food products, dishes, and beverages among girls at the beginning and end of the study. In the case of, on average, the most frequently consumed foods, such as milk, yogurt, fatty cheeses, wheat bread, poultry meat, cold cuts, fried dishes, fruit, vegetables, butter, and sweets, the values from the indications of several times a week and every day were summed. In the case of foods less frequently consumed, such as cottage cheeses, whole-grain bread, fish, white rice and pasta, eggs, fish, fast food, fruit juices, and carbonated drinks, the data from the indication of once a week were adopted.

**Figure 5:**
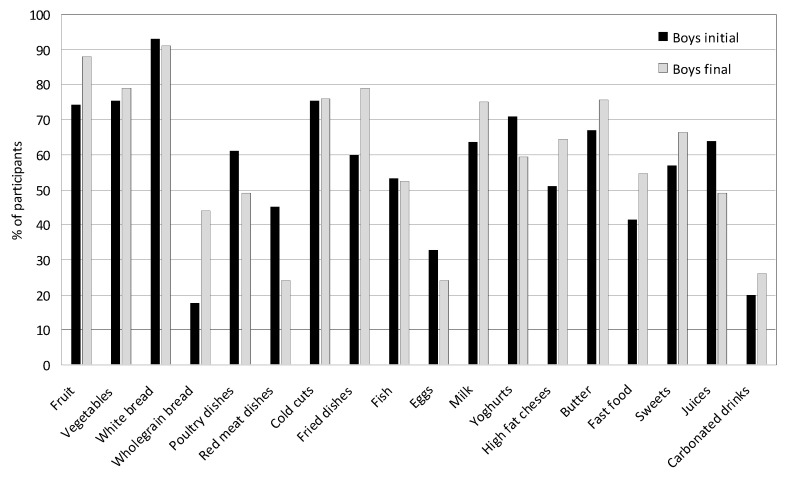
The average consumption of selected food products, dishes, and beverages among boys at the beginning and end of the study. In the case of, on average, the most frequently consumed foods, such as milk, yogurt, fatty cheeses, wheat bread, poultry meat, cold cuts, fried dishes, fruit, vegetables, butter, and sweets, the values from the indications of several times a week and every day were summed. In the case of foods less frequently consumed, such as cottage cheeses, whole-grain bread, fish, white rice and pasta, eggs, fish, fast food, fruit juices, and carbonated drinks, the data from the indication of once a week were adopted.

After four years, significant changes were noted in the consumption of food products, dishes and beverages in both the group of girls and boys. Figure 4 and Figure 5 present the average consumption at the beginning and at the end of the study of selected food products, dishes, and beverages in the group of girls and boys. In the case of on average most frequently consumed foods, such as milk, yogurt, fatty cheeses, wheat bread, poultry meat, cold cuts, fried dishes, fruit, vegetables, butter, and sweets, the values from the indications of several times a week and every day were summed. In the case of foods less frequently consumed, such as cottage cheeses, whole-grain bread, fish, white rice and pasta, eggs, fish, fast food, fruit juices, and carbonated drinks, the data from the indication of once a week were adopted.

Girls most often consumed four meals a day (37.34%). Just 32.00% of girls consumed five meals and 25.00% consumed three meals a day. Compared to the initial measurement, there was an increase in the consumption of three meals from 16.83% to 33.20% (*p* = 0.025) and a decrease in the consumption of five meals (from 45.54% to 18.45%; *p* = 0.001). At the beginning of the study, 70.25% of girls ate breakfast, and at the end, 64.36% ate breakfast (*p* = 0.070). After four years, there was a decrease in snacking between meals from 91.09% to 81.06% (*p* = 0.088). Sweets and salty snacks between meals were eaten by 27.25% of girls, and fruit by 29.50% of girls. Throughout the study period, the consumption of salty snacks chosen between main meals increased.

In girls, a significant increase was observed in the consumption of fruit (from 78.21% to 92.00%; *p* = 0.001), whole-grain bread (from 32.67% to 45%; *p* = 0.031), fried meat and flour dishes (from 63% to 72%; *p* = 0.041), and fatty cheeses (from 47.50% to 61.25%; *p* = 0.021) several times a week, and fast food once a week (from 25.74% to 45.50%; *p* = 0.031) (Figure 4). There was also a slight increase in the consumption of milk (from 69.30% to 70.13%; *p* = 0.101), vegetables (from 87.13% to 81.50%; *p* = 0.091), butter (from 73.50% to 75%; *p* = 0.079), sweets (from 70% to 73%; *p* = 0.110), and carbonated drinks (from 27% to 29%; *p* = 0.970). The consumption of yogurts decreased (from 69.50% to 52.50%; *p* = 0.021); poultry meat (from 44.50% to 40%; *p* = 0.189), red meat (from 19.80% to 12.22%; *p* = 0.097), cold cuts and sausages (from 70.29% to 51.28%; *p* = 0.001), eggs (from 21.78% to 17.52%; *p* = 0.065), and juices (from 49.50% to 42.50%; *p* = 0.091) decreased to several times a week and fish to once a week (from 64.36% to 42.36%; *p* = 0.000). The consumption of wheat bread several times a week was high both at the beginning and at the end of the study and amounted to 92.00%.

In girls, wheat bread was frequently eaten both at the beginning and at the end of the study, with an increase in its consumption (from 66.34% to 72.28%; *p* = 0.001). The daily consumption of wheat bread was still higher compared to wholemeal bread, with the daily consumption of wholemeal bread increasing from 13.86% to 23.50% (*p* = 0.140). The consumption of fine-grained groats and coarse-grained groats several times a week decreased from 43.56% to 24.04% (*p* = 0.001) and from 36.63% to 21.80% (*p* = 0.019), respectively. In girls, a decrease in the declared consumption of cold cuts and sausages was observed from 30.69% to 24.00% (*p* = 0.071). Poultry meat was still consumed more often than red meat. However, there was a decrease in the consumption of poultry meat dishes from 44.55% to 40.00% (*p* = 0.189). Compared to the beginning of the study, the consumption of red meat decreased by almost 7.90% and poultry meat by 4.80%. Both at the beginning and the end of the study, fruits and vegetables were usually consumed every day, with an increase in their consumption. Daily fruit consumption increased from 40.59% to 69.00% (*p* = 0.001), and vegetables from 53.47% to 62% (*p* = 0.036). Daily butter consumption increased from 39.50% to 53.00% (*p* = 0.040). The consumption of fast food once a week increased significantly, from 25.74% to 45.50% (*p* = 0.031). The consumption of sweets every day increased from 41.00% to 43.50% (*p* = 0.072). Juices were usually drunk once a week, with a decrease in their consumption from 49.50% to 42.50% (*p* = 0.091). The consumption of carbonated drinks increased from 27.10% to 29.20%, and the consumption of energy drinks remained very low.

Boys most often consumed four meals a day (40.51%); 28.00% of boys consumed five meals a day and 27.12% consumed three meals. Compared to the beginning of the study, an increase in the consumption of three meals was noted from 21.24% to 33% (*p* = 0.032) and a decrease in the consumption of five meals a day was noted (from 32.74% to 23%; *p* = 0.091). At the beginning of the study, 68.00% of boys ate breakfast, while at the end 59.53% ate breakfast (*p* = 0.041). After four years, there was a decrease in snacking between meals from 92.04% to 83.00% (*p* = 0.171). Fruit between meals was eaten by 36.00% of boys and salty snacks by 24.50%. Compared to the initial period, the consumption of fruit and salty snacks between the main meals increased.

In boys, an increase in the consumption of fruit (from 74.33% to 88%; *p* = 0.081), whole-grain bread (from 17.69% to 44.00%; *p* = 0.001), fried meat and flour dishes (from 60.00% to 79.10%; *p* = 0.081), fatty cheeses (from 51.00% to 64.50%; *p* = 0.091), and fast food (from 41.59% to 55.00%; *p* = 0.011) was observed (Figure 5). There was also an increase in the consumption of milk (from 63.71% to 75%; *p* = 0.042), vegetables (from 75.22% to 79%; *p* = 0.191), butter (from 67% to 75.50%; *p* = 0.049), cold cuts and sausages (from 75.22% to 76%; *p* = 0.778), sweets (from 57% to 66.50%; *p* = 0.010), and carbonated drinks (from 20,25% to 26.50%; *p* = 0.780). The consumption of wheat bread was both high at the beginning and at the end of the study and amounted to 92.20%. The consumption of fermented milk drinks decreased (from 71% to 59.50%; *p* = 0.001), as well as the consumption of white meat (from 61.06% to 49%; *p* = 0.019), red meat (from 45.13% to 24%; *p* = 0.017), eggs (from 32.74% to 24.20%; *p* = 0.005), fish (from 53.10% to 52.38%; *p* = 0.370), and juices (from 64.00% to 49.10%; *p* = 0.001).

In boys, the consumption of milk (from 23.89% to 32.00%; *p* = 0.040) and fatty cheeses (from 13.50% to 20.50%; *p* = 0.030) increased, while the consumption of yogurt (from 17.50% to 13.50%; *p* = 0.132) and cottage cheese (from 14.50% to 7%; *p* = 0.021) decreased.

Wheat bread was often consumed both at the beginning and at the end of the study, with an increase in its daily consumption from 76.99% to 82.20% (*p* = 0.061). The daily consumption of wholemeal bread increased from 6.19% to 18.00% (*p* = 0.010). The consumption of fine-grained groats several times a week decreased from 40.71% to 28.00% (*p* = 0.022) and coarse-grained groats increased from 20.35% to 34.00% (*p* = 0.034). An increase in the daily consumption of cold cuts and sausages was observed from 32.74% to 50.00% (*p* = 0.001). Poultry meat was still consumed more often than red meat (55.03% vs. 34.56%, respectively; *p* = 0.001). After four years, a significant decrease in the consumption of poultry meat (a decrease of 12.00%) and red meat (a decrease of 20.10%) was noted. An increase in the daily consumption of fried meat or flour dishes was noted (from 15.00% to 27.20%; *p* = 0.001). Egg consumption decreased by 7.90% and fish consumption by 9.00%. Both at the beginning and at the end of the study, fruits and vegetables were consumed every day, with an increase in their consumption. Daily fruit consumption was declared by 34.51% of boys at the beginning of the study and 58.00% at the end (*p* = 0.000), and daily vegetable consumption was declared by 38.05% and 55.00%, respectively (*p* = 0.006). Daily butter consumption increased from 37.50% to 59.00% (*p* = 0.000). Daily sweets consumption increased from 34.00% to 39.20% (*p* = 0.002).

**Figure 6:**
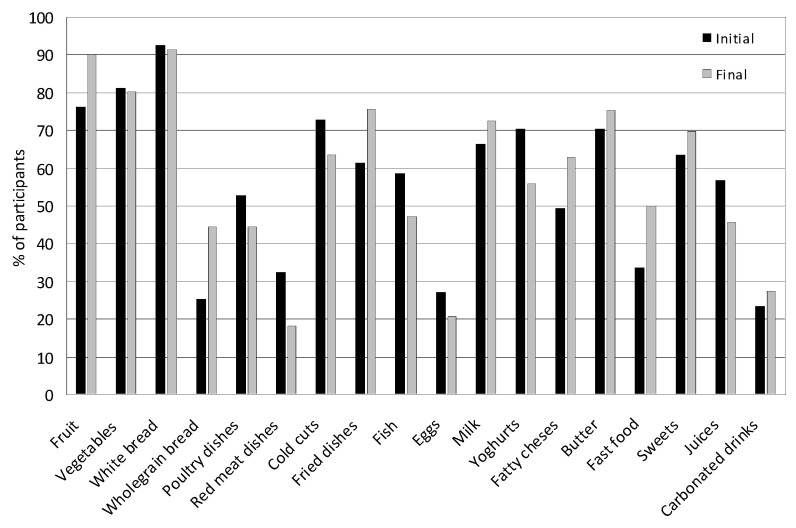
The average consumption of selected food products, dishes, and beverages in the entire group at the beginning and end of the study. In the case of, on average, the most frequently consumed foods, such as milk, yogurt, fatty cheeses, wheat bread, poultry meat, cold cuts, fried dishes, fruit, vegetables, butter, and sweets, the values from the indications of several times a week and every day were summed. In the case of foods less frequently consumed, such as cottage cheeses, whole-grain bread, fish, white rice and pasta, eggs, fish, fast food, fruit juices, and carbonated drinks, the data from the indication of once a week were adopted.

Figure 6 presents the average variability of children’s nutritional behaviors at the beginning and at the end of the study, regardless of gender. After four years, there was a significant increase in the mean consumption of fruit (from 76.27% to 90.00%; *p* = 0.026), whole-grain bread (from 25.18% to 44.50%; *p* = 0.001), and milk (from 66.51% to 72.57%; *p* = 0.078), i.e., products that should be consumed more often in a healthy diet. In addition, there was an increase in the consumption of products that should be limited in a healthy diet, such as fried flour and meat dishes (from 61.50% to 75.50%; *p* = 0.450), fatty cheeses (from 49.25% to 62.87%; *p* = 0.021), butter (from 70.25% to 75.25%; *p* = 0.142), fast food (from 33.66% to %; *p* = 0.041), sweets (from 63.50% to 69.75%; *p* = 0.141), and carbonated drinks (from 23.50% to 27.50%; *p* = 0.241). An unfavorable decrease was noted in the consumption of coarse-grained groats (from 28.49% to 27.90%; *p* = 0.247), vegetables (81.17% to 80.25%; *p* = 0.278), poultry meat (from 52.80% to 44.50%; *p* = 0.025), fish (from 58.73% to 47.37%; *p* = 0.092), eggs (from 27.26% to 20.76%; *p* = 0.419), and yogurts (from 70.25% to 56%; *p* = 0.001). Also, the mean consumption of red meat (from 32.46% to 18.11%; *p* = 0.047), cold cuts (from 72.75% to 63.64%; *p* = 0.097), and fruit juices (from 56.75% to 45.75%; *p* = 0.741) decreased, i.e., products whose consumption should be limited in a healthy diet.

### 3.7. PCA

**Figure 7:**
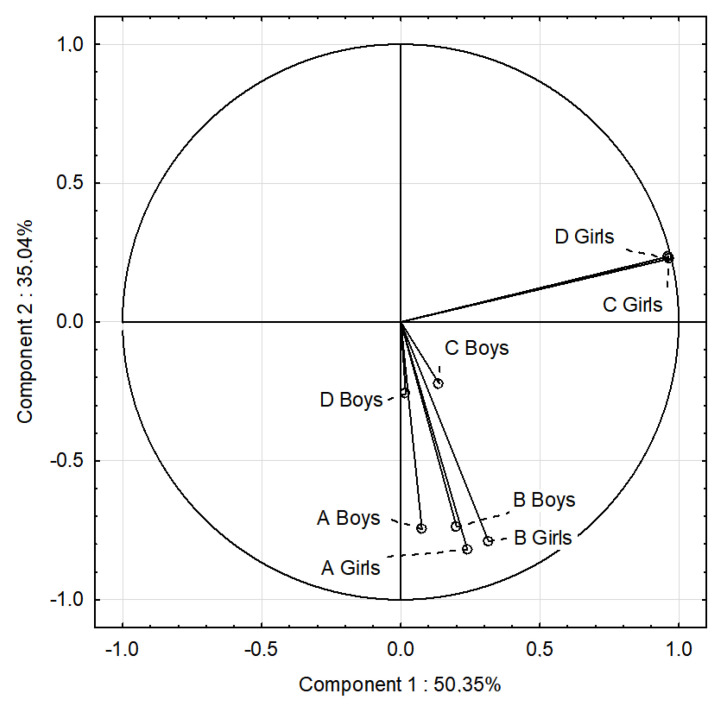
PCA for nutritional behaviors, BMI, and FM of girls and boys at the beginning of the study. A Girls—favorable nutritional behaviors of girls; B Girls—unfavorable nutritional behaviors of girls; C Girls—BMI of girls; D Girls—FM of girls; A Boys—favorable nutritional behaviors of boys; B Boys—unfavorable nutritional behaviors of boys; C Boys—BMI of boys; D Boys—FM of boys.

**Figure 8:**
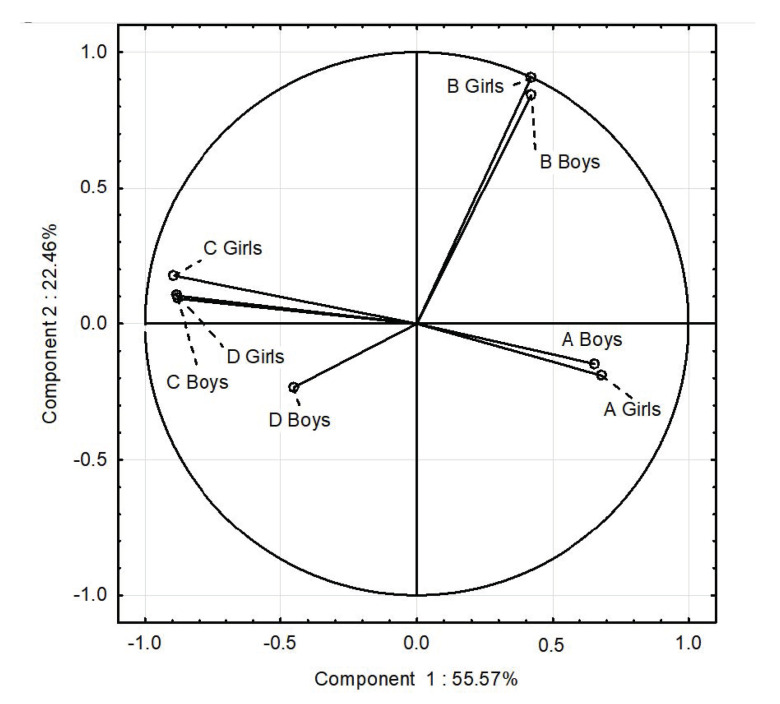
PCA for nutritional behavior, BMI, and FM of girls and boys at the end of the study. A Girls—favorable nutritional behaviors of girls; B Girls—unfavorable nutritional behaviors of girls; C Girls—BMI of girls; D Girls—FM of girls; A Boys—favorable nutritional behaviors of boys; B Boys—unfavorable nutritional behaviors of boys; C Boys—BMI of boys; D Boys—FM of boys.

The influence of gender on favorable nutritional behaviors (the average consumption of products such as vegetables, fruits, whole grains, coarse-grained groats, cottage cheese, yogurts, milk, poultry meat, eggs, and water, which are recommended in a healthy diet) and unfavorable nutritional behaviors (the average consumption of wheat bread, fine-grained groats and wheat pasta, rice, fried meat and flour dishes, red meat, cold cuts, canned meat, fatty cheeses, sweets, fast food, carbonated drinks, and juices), BMI and FM at the beginning and end of the study were additionally evaluated by multivariate cluster analysis (Figure 7 and Figure 8).

Figure 7 presents the analyzed parameters and nutritional behavior at the beginning of the study. The PCA graph demonstrated that the nutritional behaviors of girls and boys were positively correlated, while the BMI and FM of girls and boys were negatively correlated. In girls, a significant increase in BMI and FM was noted, which was associated with an increase in unfavorable nutritional behaviors. In boys, an increase in favorable nutritional behaviors influenced a decrease in BMI and FM.

Figure 8 shows the analyzed parameters and nutritional behavior at the final measurements. At the end of the study, the nutritional behavior of girls and boys was similar, but an increase in BMI and FM was observed in girls. An increase in BMI was associated with higher FM values and unfavorable nutritional behaviors in girls and boys. At the end of the study, significant differences were noted in nutritional behaviors, BMI and FM. A positive correlation was demonstrated for nutritional behaviors, while a negative correlation was demonstrated for BMI and FM. In girls, an increase in unfavorable nutritional behaviors correlated more strongly with an increase in BMI and FM than at the beginning of the study. In boys, an increase in unfavorable nutritional behaviors correlated more strongly with an increase in BMI than FM. More favorable nutritional behaviors in boys resulted in a lower FM.

### 3.8. Cluster Analysis

**Figure 9:**
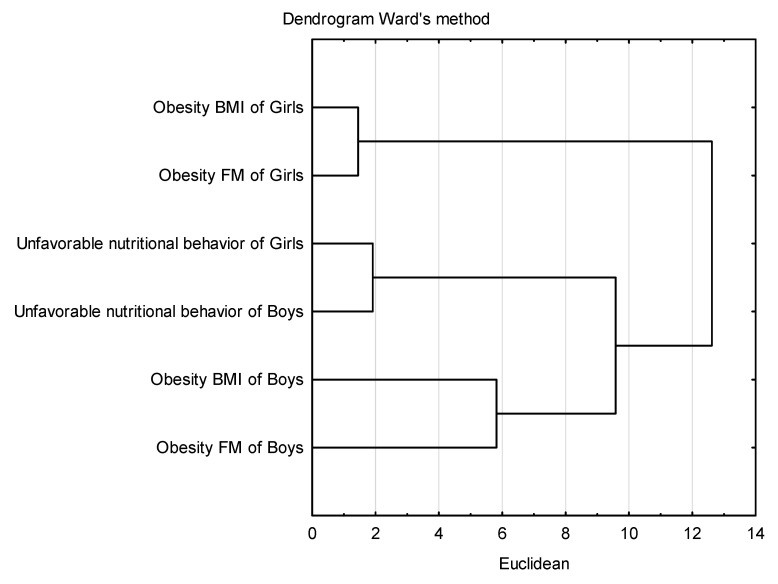
Cluster analysis of unfavorable nutritional behaviors, BMI, and FM obesity in girls and boys.

Figure 9 shows the average values of unfavorable nutritional behaviors and obesity analyzed based on the FM and BMI percentile charts. The separated clusters showed groups of similar nutritional behaviors, BMI, and FM. Three clusters were separated, the first cluster included obesity BMI and FM of girls, the second cluster included unfavorable nutritional behaviors of girls and boys, and the third cluster included obesity BMI and FM of boys. Girls were characterized by higher BMI and FM values. A strong correlation was noted between BMI and FM of girls and boys with obesity, the decrease in unfavorable nutritional behaviors affected the reduced rate of obesity FM and BMI, especially in boys.

## 4. Discussion

This study showed an increase in the levels of overweight and obesity with age, regardless of gender. The period of our research overlapped unintentionally with the COVID-19 pandemic. Many studies have shown that the pandemic caused people to gain weight and change their eating habits [1,3].

The hypothesis regarding differences in eating behavior, BMI, and FM between the sexes was confirmed. We observed an increase in excess body weight and adipose tissue with age, especially in girls, which is consistent with the working hypothesis. Despite more favorable eating behaviors in girls, we observed a significant increase in their body weight, which can be explained by hormonal changes and puberty.

Girls were, on average, more overweight and obese than boys throughout the study period. Nutritional behaviors were similar in both girls and boys. After four years, the frequency of unfavorable nutritional behaviors increased significantly in the whole group. We assumed that with age, there would be an increase in more beneficial eating behaviors, but the outbreak of the pandemic will undoubtedly affect the obtained results. This is consistent with previous studies, which also showed that nutritional behaviors, BMI, and FM changed with the age of children [26,30,44,45].

The BMI was similar between girls and boys; at the beginning of the study, it was on average 18.34 kg/m^2^ and at the end of the study it was 21.29 kg/m^2^. Lower BMI values were observed in the study by Deren et al. (2020) [46]. They showed that the average BMI of children aged 10 was 17.40 kg/m^2^ (17.10 kg/m^2^ in girls; 17.70 kg/m^2^ in boys) and at the age of 14, it was higher compared to our observations and amounted to 19.25 kg/m^2^ (19.50 in girls; 19.50 in boys) [46]. However, higher BMI values in children aged 14 were noted in the study by Vehrs et al. (2022) [47]. The prevalence of overweight and obesity in school-aged children is high [48]. Our results also support these observations. During the entire study period, 11.52% of children were obese (12.53% of girls; 10.50% of boys), 14.45% were overweight (15.76% of girls; 13.12% of boys), 10.67% were underweight (10.74% of girls; 10.61% of boys), and 63.36% were normal weight (60.96% of girls; 65.76% of boys). On average, about 25.00% of children were overweight. Our values are higher than the global data showing that 22.20% of children and adolescents under 18 years of age are overweight [44]. We found that girls were more overweight than boys (28.29% of girls; 23.63% of boys). A different observation was made by Deren et al. (2020), where the incidence of overweight was significantly higher in boys than in girls [46]. Furthermore, in the cited work, it was observed that, regardless of gender, the percentage of children with normal body weight increased with age [46]. After four years, we observed an increase in BMI in both boys and girls. We observed that, in girls, an increase in BMI was associated with an increase in FM, and in boys, an increase in BMI was not associated with an increase in FM. Our results are consistent with the results of Stierman et al. (2021) [45]. These results confirmed that the increase in BMI with age in boys is mainly attributed to an increase in lean mass, while in girls, it is mainly due to an increase in body fat mass [45]. Our study used data collected before and after the COVID-19 pandemic ended. Recent studies demonstrated the impact of the pandemic on the increase in obesity share in school-aged children, mainly due to remote learning, increased screen time, and reduced PA and other sedentary behaviors [49]. In our study, between the initial measurement (before the pandemic) and the final measurement (just after ending), an increase in the percentage of children with excess body weight (overweight and obesity) was observed in girls from 27.09% to 29.50% and in boys from 21.26% to 26.00%. Similar results were obtained by Lartey et al. (2023), who showed a significant increase in the percentage of children who were obese [50]. Woolford et al. (2021) also reported a significant increase in excess body weight during the COVID-19 pandemic [51].

Body fat content changes with age and also depends on gender. During puberty, boys experience a decrease in body fat due to a faster increase in lean mass, whereas in girls, an increase in body fat is observed. The higher and increasing amount of adipose tissue in school-aged girls is physiological and results from puberty in this period. Our study also noted a higher fat mass content in girls (23.17% of girls; 16.20% of boys). The above findings are consistent with other studies [18,52].

According to the recommendations, the body fat mass should be lower than 30.00% in girls and lower than 22.50% in boys [18]. In our study, the mean body fat mass was 23.17% in girls and 16.20% in boys. The body fat mass expressed in kg in girls was 8.73 kg at the beginning of the study and 14.88 kg at the end, and in boys 7.49 kg and 9.87 kg, respectively. Girls had, on average, a higher body fat mass expressed in kilograms than boys (11.80 kg and 8.68 kg, respectively; *p* = 0.001). In the study by Shypailo et al. (2020), the average values of body fat mass for children aged 10–12 were similar and amounted to 11.40 kg in girls and 10.00 kg in boys, while for children aged 13–15, it amounted to 15.60 kg and 14.50 kg, respectively [53]. At the end of the study, the percentage of body fat mass was 24.57% in girls and 14.60% in boys. Similar results were obtained in the study by Vehrs et al. (2022), where the percentage of body fat mass in 14-year-old boys was 14.10%, and in girls, it was higher compared to our observations and amounted to 29.40% [47]. During the study, an increase in body fat mass was noted in girls and a decrease in boys. In girls, there was a mean increase in FN from 21.77% to 24.57%, and in boys, a decrease from 17.80% to 14.60%. Similarly, Stierman et al. (2021) reported a decrease in the percentage of body fat mass in boys around the age of 11, while in girls, there was an increase [45]. Zhao et al. (2023) showed that the percentage of body fat mass in girls increased significantly between the ages of 11 and 15 years, and in boys aged 11–14 years, it tended to decrease [54]. Our data are consistent with previous reports that, although BMI and FM values were similar in boys and girls, there were clear gender differences in the percentage of children with excess body weight [55,56].

It was shown that, with age, the percentage of children with unfavorable nutritional behaviors increased in both girls and boys. Many studies confirm that following a Mediterranean diet can help maintain a healthy body weight and prevent obesity early in life [30]. Only a small number of the children showed daily intake of whole-grain products, vegetables, fruits, yogurts, and cottage cheeses. An even smaller percentage of the study participants consumed fish, legumes, coarse-grained groats, oatmeal, and whole-grain bread, which could have contributed to higher BMI and FM. The unfavorable nutritional behaviors shown in this study were also noted in the works of other authors [23,57,58]. As in this work, Lytle et al. (2000) showed that the consumption of breakfast, fruit, vegetables, and milk decreased with age [23].

Throughout the study period, we observed a high consumption of fried meat and flour dishes, fatty cheeses, butter, sweets, and fruit juices. The consumption of the above products increased with the age of the children, which likely had a significant impact on body mass and body fat content. With age, the consumption of fast food and sweets increased significantly in both boys and girls. The frequent consumption of sweets, fast food, and carbonated drinks is one of the most harmful nutritional behaviors among children and adolescents. Our study showed that participants most often consumed fast food once a week (35.62% of girls; 48.05% of boys), while sweets were most often consumed every day (42.25% of girls; 36.25% of boys). Similar results were observed in the work of Scaglioni et al. (2018), where 41.00% of teenagers consumed fast food once a week [59]. Our study showed a moderate consumption of carbonated drinks, and to our surprise, the consumption of carbonated drinks decreased at the age of 14. Jakobsen et al. (2023) showed that high consumption of sweetened carbonated drinks and fast food was the main risk factor for overweight [60]. It is also suggested that school-aged children who eat a lot of processed foods are more likely to become overweight and obese [11,12,13]. Contrary to dietary recommendations, fruits were more often consumed than vegetables, and wheat bread was more often consumed than whole grains. Additionally, fine-grained groats, white rice, and wheat pasta were more often eaten compared to oatmeal, whole-grain pasta, and coarse-grained groats. Similarly, Hur et al. (2012) found that children and adolescents do not consume enough whole-grain products [61]. Jakobsen et al. (2023) demonstrated that higher consumption of meat and wheat bread was associated with an increased risk of overweight and obesity, while higher consumption of whole-grain products was associated with reduced risk [60].

Our study demonstrated that girls consumed milk, yogurt, whole-grain bread, fruit, and vegetables more often than boys. This was consistent with the findings of Hamulka et al. (2018), who showed that girls had greater nutritional knowledge than boys [57]. Boys were more likely to consume poultry meat, red meat, cold cuts and sausages, and eggs than girls. It is worth mentioning that girls were more likely to consume sweet and salty snacks than boys.

Banfield et al. (2016) and Winpenny et al. (2018) demonstrated that the quality of the diets of adolescents is less favorable than that of younger children [62,63]. Our observations are in agreement with the above. After four years, we observed a decrease in the consumption of coarse-grained groats, eggs, poultry and red meat, fish, and cottage cheese. However, the consumption of fast food, fried meat, flour dishes, butter, and fatty cheeses increased. The deterioration of nutritional behaviors in the study group probably influenced the increase in BMI and body fat.

Although our study yielded many observations, it also had some limitations. First, the study was limited to a relatively small group of participants. The study could be further extended to include teenagers of a different age and from a wider geographical area. The questionnaire only included a question about the frequency of consumption of various food products, without taking into consideration the quantity. The data on nutritional behaviors were based solely on children’s subjective declarations. Another limitation was the anonymity of the study, which made it impossible to compare changes in nutritional behaviors of individual children and correlates with BMI and FM. The influence of the economic situation on the formation of lifestyle and nutritional behaviors is often suggested, but this aspect was not included in this study. The study was conducted during the unexpected outbreak of the COVID-19 pandemic, which impacted the research program. The authors acknowledge that lockdown restrictions may have had an impact on the body weight, body fat content, and eating behavior of children, and therefore, we can expect a large deviation. There should be more physical education lessons in primary schools and regular physical activity, which has a positive effect on children’s body weight and body fat content and may help to create more favorable eating behaviors. Further studies should be focused on analyzing PA in addition to nutritional behavior and body weight.

Childhood obesity is a public health problem that is growing worldwide. It is recommended to promote healthy diets and the regular monitoring of the body fat content of school-aged children.

## 5. Conclusions

In the studied group of children, there were significant differences in BMI, FM, and nutritional behaviors between girls and boys, as well as between the ages of 10 and 14. With age, an increase in the frequency of excessive body weight was observed, particularly in girls. Body fat mass was significantly higher at the end of the study, especially in girls, while body fat mass decreased in boys. Girls were characterized by a higher percentage of overweight and obesity interpreted based on percentile charts of body fat content and BMI. The children showed moderate nutritional behaviors, but with age, unfavorable nutritional behaviors intensified, especially in boys. Despite this, girls were more willing to eat sweets and salty snacks than boys. During the study, an increase in body fat mass was noted in girls and a decrease in boys. On average, in the studied group an increase in BMI was associated with an increase in body fat mass in girls but not in boys.

With age, in the evaluated group, unfavorable nutritional behaviors and excessive body weight levels increased. More extreme levels of overweight and obesity and higher body fat contents were found in girls than boys. Although girls’ nutritional behaviors were healthier, they were at a higher risk of excessive body weight. The increased promotion of a healthy diet and the regular monitoring of body fat content in school-aged children is strongly recommended.

## Figures and Tables

**Table 1:** Comparison of children’s anthropometric indicators between initial and final measurements.

Indicator	Initial				Final				Total *p*
	Total	Boys	Girls	*p*	Total	Boys	Girls	*p*	
Height (cm)	142.96	143.28	142.68	0.041	167.23	169.76	164.67	0.021	0.001
Weight (kg)	37.80	38.15	37.46	0.070	59.90	62.58	57.19	0.041	0.000
BMI (kg/m^2^)	18.34	18.38	18.28	0.120	21.29	21.53	21.06	0.060	0.025
BMI (percentile)	50th	50th	50th	0.830	75th	75th	75th	0.937	0.120

BMI—body mass index (kg/m^2^); percentile corresponding to BMI percentile charts; *p*—ANOVA; *p* ≤ 0.05.

**Table 2:** Percent of children in each BMI category during study period.

Indicator	Initial				Final				Total *p*
	Total Sample	Boys	Girls	*p*	Total Sample	Boys	Girls	*p*	
Underweight	10.17	10.22	10.13	0.100	11.17	11.00	11.35	0.240	0.091
Normal	65.65	68.52	62.78	0.001	61.07	63.00	59.15	0.001	0.001
Overweight	15.27	12.75	17.78	0.031	13.62	13.50	13.75	0.170	0.047
Obesity	8.91	8.51	9.31	0.640	14.12	12.50	15.75	0.001	0.000

*p*—ANOVA; *p* ≤ 0.05.

**Table 3:** Body fat (FM) % and kg.

Fat Mass, % (kg)									
Measurement Session	Average Age (Years)	Total Sample	Median	Min.	Max.	SD	95% CI	*p*	ESs
Average									
Initial	10.27	19.77 (8.09)	18.15 (6.70)	6.50 (1.70)	47.40 (40.60)	7.62	7.00–8.36	0.620	0.460
Final	13.90	19.59 (12.38)	18.65 (10.70)	3.00 (1.30)	46.20 (46.00)	8.73	7.97–9.65		
Mean from both sessions		19.68 (10.23)							
Girls									
Initial	10.27	21.77 (8.73)	21.15 (7.75)	6.50 (1.70)	37.30 (21.10)	7.56	7.50–6.65	0.040	0.510
Final	13.90	24.57 (14.88)	23.65 (12.85)	6.40 (2.80)	46.20 (46.00)	7.82	6.89–9.04		
Mean from both sessions		23.17 (11.80)							
Boys									
Initial	10.27	17.80 (7.49)	15.60 (5.70)	8.40 (2.20)	47.40 (40.60)	7.23	6.44–8.25	0.032	0.468
Final	13.90	14.60 (9.87)	14.65 (8.90)	3.00 (1.30)	34.30 (35.70)	6.46	5.69–7.47		
Mean from both sessions		16.20 (8.68)							

SD—standard deviation; 95% CI—confidence interval; *p*—*t*-test; ESs—effect sizes; Min.—minimum; Max.—maximum. Measurement sessions: initial—2017; final—2021.

**Table 4:** Percent of children in each FM category during study period.

Indicator	Initial				Final			
	Total	Boys	Girls	*p*	Total	Boys	Girls	*p*
Underweight	10.75	10.99	10.52	0.079	5.73	6.03	5.45	0.087
Normal	60.73	62.13	59.33	0.065	58.37	60.95	55.80	0.025
Overweight	17.59	16.22	18.95	0.018	22.76	20.77	24.75	0.057
Obesity	10.93	10.66	11.20	0.095	13.12	12.25	14.00	0.061

*p*—ANOVA; *p* ≤ 0.05.

**Table 5:** Average frequency of consumption of food products, dishes, and beverages, %.

No	Foods	Sex	Frequency of Consumption [%]					Statistical Measures *
			Never	1–3 Times a Month	Once a Week	Several Times a Week	Every Day	
1.	Milk	Girls	4.50	5.76	20.03	30.41	39.30	χ^2^ = 6.812, df-4, *p* = 0.146, VC = 0.178
		Boys	1.83	6.92	21.89	41.41	27.95	
2.	Yogurts (natural and flavored)	Girls	5.00	11.75	22.25	40.25	20.75	χ^2^ = 10.469, df-4, *p* = 0.033, VC = 0.221
		Boys	7.75	11.25	15.75	49.75	15.50	
3.	Cottage chesses	Girls	18.25	20.50	29.25	20.00	12.00	χ^2^ = 11.693, df-4, *p* = 0.019, VC = 0.233
		Boys	11.75	22.00	22.00	33.50	10.75	
4.	High-fat cheeses (including processed and blue cheeses)	Girls	4.13	13.75	27.75	37.25	17.13	χ^2^ = 1.241, df-4, *p* = 0.871, VC = 0.076
		Boys	4.50	16.00	21.75	40.75	17.00	
5.	Wheat bread and rolls	Girls	0.61	1.75	5.71	22.62	69.31	χ^2^ = 3.389, df-4, *p* = 0.044, VC = 0.125
		Boys	0.50	1.25	6.25	12.46	79.50	
6.	Whole-grain bread and rolls	Girls	13.20	14.21	33.76	20.16	18.68	χ^2^ = 13.284, df-4, *p* = 0.010, VC = 0.249
		Boys	13.46	22.06	33.62	18.75	12.10	
7.	White rice, white pasta, fine-grained groats	Girls	2.75	9.41	54.04	25.10	8.71	χ^2^ = 4.956, df-4, *p* = 0.291, VC = 0.152
		Boys	2.77	8.65	54.22	24.27	10.08	
8.	Coarse-grained groats, buckwheat groats, oatmeal	Girls	4.53	13.54	52.71	22.52	6.70	χ^2^ = 8.087, df-4, *p* = 0.008, VC = 0.194
		Boys	5.15	11.92	55.74	21.35	5.83	
9.	Poultry meat	Girls	2.79	14.41	40.50	19.92	22.38	χ^2^ = 8.734, df-4, *p* = 0.048, VC = 0.202
		Boys	1.00	8.98	34.99	28.23	26.80	
10.	Red meat	Girls	6.76	27.71	49.52	9.08	6.93	χ^2^ = 19.835, df-4, *p* = 0.000, VC = 0.304
		Boys	6.21	14.25	44.97	17.96	16.60	
11.	Cold cuts, sausages, frankfurters	Girls	9.27	9.43	20.53	33.44	27.33	χ^2^ = 1.141, df-4, *p* = 0.887, VC = 0.703
		Boys	4.50	5.10	14.79	34.24	41.37	
12	Canned meats	Girls	65.00	25.75	9.25	0.00	0.00	χ^2^ = 12.467, df-4, *p* = 0.614, VC = 0.241
		Boys	64.25	22.25	8.25	5.25	0.00	
13.	Fried flour or mest meals	Girls	0.00	7.00	26.25	43.75	23.00	χ^2^ = 2.295, df-4, *p* = 0.681, VC = 0.103
		Boys	0.00	3.75	27.00	48.25	21.00	
14.	Eggs	Girls	5.72	15.98	58.65	19.04	0.61	χ^2^ = 10.796, df-4, *p* = 0.028, VC = 0.224
		Boys	3.50	9.15	58.97	24.99	3.38	
15.	Fish	Girls	20.09	23.45	53.36	2.61	0.50	χ^2^ = 7.496, df-4, *p* = 0.111, VC = 0.187
		Boys	14.07	29.21	52.74	3.10	0.88	
16.	Legumes	Girls	35.25	43.75	12.00	8.50	0.50	χ^2^ = 12.628, df-4, *p* = 0.621, VC = 0.110
		Boys	35.00	46.50	12.50	5.25	0.75	
17.	Fruit	Girls	0.00	2.48	12.42	30.56	54.00	χ^2^ = 1.357, df-4, *p* = 0.035, VC = 0.079
		Boys	1.25	5.73	11.85	34.91	46.26	
18.	Vegetables	Girls	2.24	0.00	13.45	26.83	57.48	χ^2^ = 6.933, df-4, *p* = 0.021, VC = 0.180
		Boys	2.25	2.25	18.12	30.58	46.53	
19.	Butter as an addition to bread, dishes, for frying, baking, etc.	Girls	6.25	9.75	9.75	27.75	46.50	χ^2^ = 9.075, df-4, *p* = 0.049, VC = 0.205
		Boys	3.75	4.50	20.50	23.00	48.25	
20.	Lard as an addition to bread, dishes, for frying, baking, etc.	Girls	93.75	6.25	0.00	0.00	0.00	χ^2^ = 7.824, df-4, *p* = 0.098, VC = 0.191
		Boys	90.25	6.00	2.75	1.00	0.00	
21.	Fast food, different types	Girls	5.23	52.43	35.62	5.74	0.99	χ^2^ = 6.322, df-4, *p* = 0.016, VC = 0.171
		Boys	2.77	44.36	48.05	4.38	0.44	
22.	Sweets, candies, chocolate, bars	Girls	0.75	6.50	20.75	29.75	42.25	χ^2^ = 6.256, df-4, *p* = 0.040, VC = 0.172
		Boys	1.75	9.50	27.00	25.50	36.25	
23.	Juices	Girls	12.00	19.50	46.00	14.00	8.50	χ^2^ = 7.614, df-4, *p* = 0.106, VC = 0.188
		Boys	6.25	16.25	56.50	13.50	7.50	
24.	Sweetened carbonated or non-carbonated drinks	Girls	21.25	39.75	28.00	7.25	3.75	χ^2^ = 5.997, df-4, *p* = 0.147, VC = 0.167
		Boys	16.50	37.25	23.00	14.50	8.75	
25.	Energy drinks	Girls	81.75	15.50	1.75	1.00	0.00	χ^2^ = 4.409, df-4, *p* = 0.353, VC = 0.143
		Boys	73.75	17.00	6.75	2.50	0.00	

* Chi squared test (χ^2^); *p*-value ≤ 0.05; df—degrees of freedom; VC—V Cramera relationship strength.

## Data Availability

The data supporting reported results are available on request from corresponding author.

## Abbreviations

The following abbreviations are used in this manuscript:

BIA	Bioelectrical Impedance Analysis
BMI	Body Mass Index
FM	Fat Mass
PCA	Principal Component Analysis
